# Development of a TaqMan Probe-Based Insulated Isothermal Polymerase Chain Reaction (iiPCR) Assay for Detection of *Fusarium oxysporum* f. sp. *cubense* Race 4

**DOI:** 10.1371/journal.pone.0159681

**Published:** 2016-07-22

**Authors:** Ying-Hong Lin, Yi-Jia Lin, Tsai-De Chang, Li-Ling Hong, Tzu-Yu Chen, Pi-Fang Linda Chang

**Affiliations:** 1 Department of Plant Medicine, National Pingtung University of Science and Technology, Pingtung, Taiwan; 2 Department of Plant Pathology, National Chung Hsing University, Taichung, Taiwan; 3 Agricultural Biotechnology Center, National Chung Hsing University, Taichung, Taiwan; 4 NCHU-UCD Plant and Food Biotechnology Center, National Chung Hsing University, Taichung, Taiwan; Bhabha Atomic Research Centre, INDIA

## Abstract

This study developed a novel and inexpensive detection method based on a TaqMan probe-based insulated isothermal polymerase chain reaction (iiPCR) method for the rapid detection of Panama disease caused by *Fusarium oxysporum* f. sp. *cubense* (Foc) race 4, which is currently among the most serious fungal vascular diseases worldwide. By using the portable POCKIT^™^ device with the novel primer set iiFoc-1/iiFoc-2, the Foc race 4 iiPCR assay (including DNA amplification and signal monitoring) could be completed within one hour. The developed Foc race 4 iiPCR assay is thus a user-friendly and efficient platform designed specifically for the detection of Foc race 4. The detection limit of this optimized Foc iiPCR system was estimated to be 1 copy of the target standard DNA as well as 1 fg of the Foc genomic DNA. This approach can serve as a rapid detection method for in planta detection of Foc race 4 in field-infected banana. It was concluded that this molecular detection procedure based on iiPCR has good potential for use as an efficient detection method.

## Introduction

Bananas and plantains, members of the *Musa* genus, are now cultivated in humid tropical areas worldwide, making them one of the most economically important and popular fruits crops in the world. However, the soil-borne fungal disease, banana Fusarium wilt (FW), commonly known as Panama disease, which is caused by *Fusarium oxysporum* f. sp. *cubense* (Foc), is a highly-lethal vascular fungal disease in banana plants. When Foc attacks a banana plant, it leads to yellowing and wilting of the older leaves that then progresses to the younger leaves until the whole plant is killed. This lethal effect makes Panama disease the major limiting factor for banana production worldwide [1].

Foc is not easy to eradicate from an infected field because the pathogen can produce thick-walled chlamydospores, which are highly resistant to fungicides or chemical fumigation [2] and can survive in infected soil as resting spores for more than 30 years [1,3–5]. One of the best means of managing FW at present is based on the breeding of FW-resistant lines [6–8], though this approach is very time-consuming [9,10]. In short, few effective, economical, environmentally safe, and curative management options are available for FW control [11].

Four races of Foc are recognized on banana based on the specificity of pathogenicity against various banana cultivars [12]. Race 1 is pathogenic to Gros Michel (genome type = AAA), while race 2 infects ABB cooking bananas, such as Bluggoe [12]. Race 4 (including tropical race 4, TR4; and subtropical race 4, ST4) affect Cavendish cultivars as well as those susceptible to race 1 and race 2 [12–14]. Foc TR4 has been found to cause severe damage to almost all presently popular cultivars. Hence, it has spread worldwide and brought about huge economic losses to the banana industry [15].

The timely eradication of any plants initially discovered to be infected by Foc and the avoidance of Foc-contaminated areas are important strategies for reducing the dissemination of the pathogen and the economic impacts of FW on the banana industry. Crucial control strategies targeting Foc race 4 could be implemented more effectively if early detection could be carried out to provide rapid, sensitive and specific test results for pathogen monitoring.

Molecular detection methods, including conventional polymerase chain reaction (PCR) [7], loop-mediated isothermal amplification (LAMP) [16], real-time PCR [10], and real-time LAMP (RealAmp) [17,18] assays, have been developed to specifically and rapidly detect Foc race 4 or Foc TR4. These detection methods offer high degrees of detection sensitivity and specificity.

Recently, insulated isothermal PCR (iiPCR), which is based on Rayleigh-Bénard convection PCR, has been described as being capable of sensitively and specifically detecting both RNA and DNA [19]. In addition, it can be performed with a single copper ring-wrapped capillary tube (R-tube^™^) heated by an isothermal heating source, making it relatively simple and inexpensive while foregoing the need for a relatively costly thermocycler [19–26].

**Fig 1:**
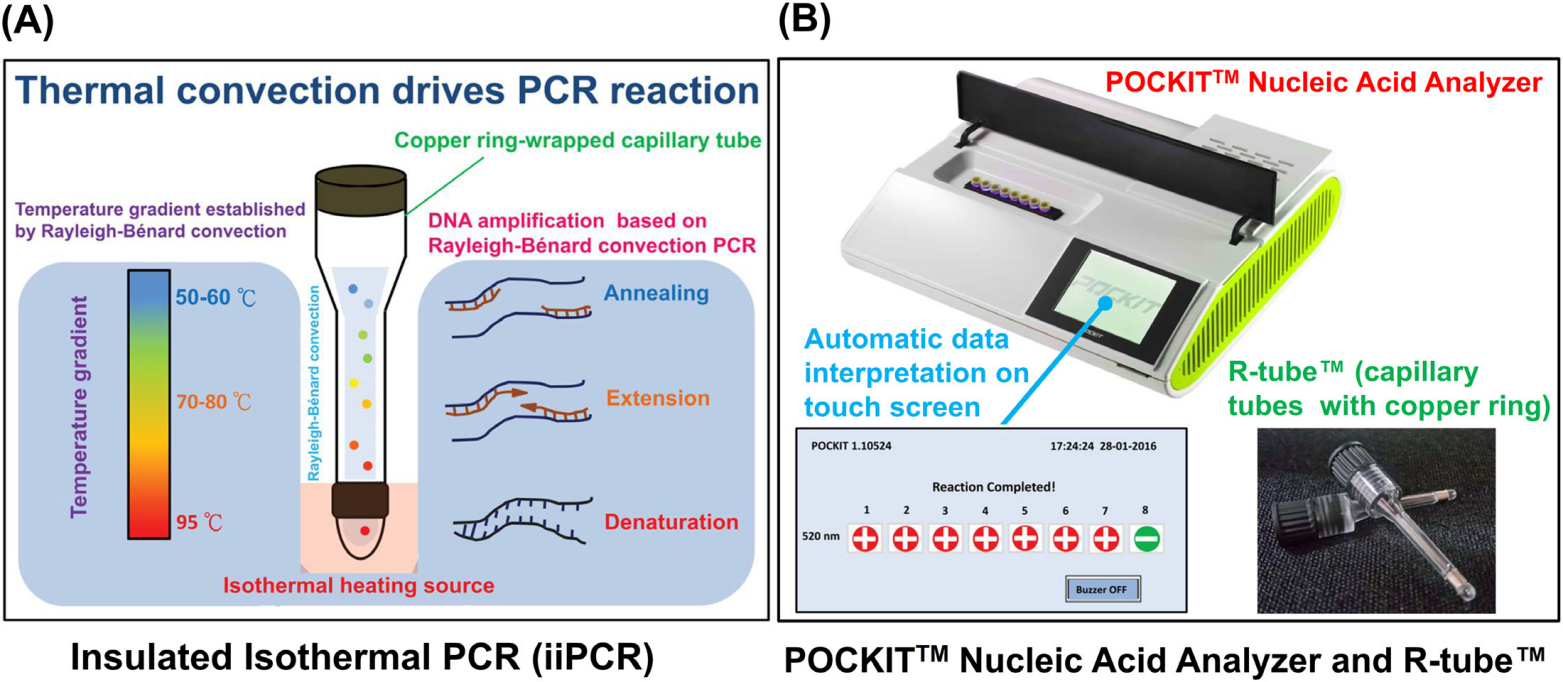
Diagrammatic representations of the insulated isothermal PCR (iiPCR) assay, the POCKIT^™^ Nucleic Acid Analyzer, and the R-tube^™^. (A) The iiPCR, established on the basis of the Rayleigh-Bénard convective PCR method, is a rapid platform for nucleic acid amplification. (B) The iiPCR system is carried out in the R-tube^™^ within the user-friendly POCKIT^™^ Nucleic Acid Analyzer designed by GeneReach Biotechnology Corporation (Taichung City, Taiwan). The POCKIT^™^ analyzer (28 × 25 × 8.5 cm, W × D × H) provides isothermal heating at the bottom of the R-tube^™^ to generate a cycling thermal convection to drive the PCR reaction. DNA amplification and product detection can be completed automatically by the POCKIT^™^ analyzer with a single default program.

The three steps of iiPCR, namely, denaturation, annealing, and extension, are completed in the copper ring-wrapped capillary tube by cycling reactions through a temperature gradient established by the Rayleigh-Bénard convection. An iiPCR instrument, the POCKIT^™^ Nucleic Acid Analyzer (GeneReach Biotechnology Corp., Taichung, Taiwan) [21,22], is now commercially available and allows the performance of a TaqMan probe-based iiPCR assay within a copper ring-wrapped capillary tube (R-tube^™^), in addition to completing the iiPCR assay automatically (Fig 1).

This paper describes a TaqMan probe-based iiPCR for rapid in planta detection of Foc race 4. The method should be sensitive, reproducible, rapid, and user-friendly, and should not require a costly thermocycler. The Foc race 4 iiPCR assay could potentially be a useful tool for routine quarantine detection of Foc race 4 to avoid further dissemination.

## Materials and Methods

### Pathogen isolates and growth condition

The Foc isolates and other isolates used in this study are listed in Table 1. All the tested Foc were isolated from banana pseudostems. A single spore culture of each tested Foc isolate was grown on a Nash-PCNB plate (1.5% peptone, 2% agar, 0.1% KH_2_PO_4_, 0.05% MgSO4·7H_2_O, 0.1% pentachloronitrobenzene, 0.03% streptomycin, and 0.1% neomycin) [27]. Other fungal isolates were grown on a potato dextrose agar (PDA) plate (200 g/l of potato extracts, 1% glucose, and 2% agar). Single colonies of *Ralstonia solanacearum* were grown on peptone sucrose agar (PSA) plate (1% peptone, 1% sucrose, 0.1% glutamic acid, pH 7.0, and 2% agar).

**Table 1:** Isolates of plant pathogens used in this study and their PCR amplification results with PCR-based identification methods.

Isolate code numbers	Diseases/species	Original hosts/tissues	Geographic locations	PCR-based identification methods used in this study			
				iiPCR^a^	ITS1/ITS4^b^	FocSc-1/FocSc-2^c^	FocTR4-F/FocTR4-R^d^
ATCC96285	Fusarium wilt of banana (FWB)/*Fusarium oxysporum* f. sp. *cubense* race 1 (Foc R1)	Banana (*Musa*. sp.) cv. Lady Finger/Pseudostem (P)	Queensland, Australia	-	+	-	-
ATCC76257	FWB/Foc race 2 (Foc R2)	Banana cv. Bluggoe/P	Honduras	-	+	-	-
ATCC38741	FWB/Foc subtropical race 4 (Foc ST4)	Banana cv. Cavendish/P	Taiwan	+	+	+	-
ATCC76262	FWB/Foc ST4	Cavendish/P	Taiwan	+	+	+	-
ATCC96289	FWB/Foc ST4	Cavendish/P	Queensland, Australia	+	+	+	-
ATCC96290	FWB/Foc ST4	Cavendish/P	Queensland, Australia	+	+	+	-
TYC-F015	FWB/Foc tropical race 4 (Foc TR4)	Cavendish/P	Kaohsiung, Taiwan	+	+	+	+
TYC-F016	FWB/Foc TR4	Cavendish/P	Kaohsiung, Taiwan	+	+	+	+
TYC-F017	FWB/Foc TR4	Cavendish/P	Kaohsiung, Taiwan	+	+	+	+
TYC-F020	FWB/Foc TR4	Cavendish/P	Kaohsiung, Taiwan	+	+	+	+
TYC-F021	FWB/Foc TR4	Cavendish/P	Kaohsiung, Taiwan	+	+	+	+
TYC-F022	FWB/Foc TR4	Cavendish/P	Pingtung, Taiwan	+	+	+	+
TYC-F023	FWB/Foc TR4	Cavendish/P	Pingtung, Taiwan	+	+	+	+
TYC-F025	FWB/Foc TR4	Cavendish/P	Pingtung, Taiwan	+	+	+	+
TYC-F026	FWB/Foc TR4	Cavendish/P	Pingtung, Taiwan	+	+	+	+
TYC-F028	FWB/Foc TR4	Cavendish/P	Pingtung, Taiwan	+	+	+	+
TYC-F029	FWB/Foc TR4	Cavendish/P	Pingtung, Taiwan	+	+	+	+
TYC-F030	FWB/Foc TR4	Cavendish/P	Pingtung, Taiwan	+	+	+	+
TYC-F031	FWB/Foc TR4	Cavendish/P	Pingtung, Taiwan	+	+	+	+
TYC-F032	FWB/Foc TR4	Cavendish/P	Pingtung, Taiwan	+	+	+	+
TYC-F033	FWB/Foc TR4	Cavendish/P	Pingtung, Taiwan	+	+	+	+
TYC-F034	FWB/Foc TR4	Cavendish/P	Pingtung, Taiwan	+	+	+	+
TYC-F035	FWB/Foc TR4	Cavendish/P	Pingtung, Taiwan	+	+	+	+
TYC-F036	FWB/Foc TR4	Cavendish/P	Pingtung, Taiwan	+	+	+	+
TYC-F037	FWB/Foc TR4	Cavendish/P	Pingtung, Taiwan	+	+	+	+
TYC-F038	FWB/Foc TR4	Cavendish/P	Pingtung, Taiwan	+	+	+	+
TYC-F039	FWB/Foc TR4	Cavendish/P	Pingtung, Taiwan	+	+	+	+
TYC-F040	FWB/Foc TR4	Cavendish/P	Pingtung, Taiwan	+	+	+	+
TYC-F027	FWB/Foc race 1	Banana cv. Latundan/P	Pingtung, Taiwan	-	+	-	-
TYC-F003	Alternaria speckle of banana/*A*. *alternata* (Aa)	Cavendish/Leaf (L)	Pingtung, Taiwan	-	+	-	-
TYC-F005	Alternaria speckle of banana/Aa	Cavendish/L	Pingtung, Taiwan	-	+	-	-
YJC-F007	Cladosporium speckle of banana/*Cladosporium musae*	Cavendish/L	Pingtung, Taiwan	-	+	-	-
YJC-F004	Crown rot of banana/*Botryosphaeria dothidea*	Cavendish/Fruit (F)	Tainan, Taiwan	-	+	-	-
TYC-F013	Fruit rot of banana/*F*. *subglutinans*	Cavendish/F	Kaohsiung, Taiwan	-	+	-	-
TYC-F014	Fruit rot of banana/*F*. *sonani*	Cavendish/F	Kaohsiung, Taiwan	-	+	-	-
YJL-F036	Anthracnose of banana/*Colletotrichum gloeosporioides*	Cavendish/L	Pingtung, Taiwan	-	+	-	-
LNH-F001	Cordana leaf spot of banana/*Cordana musae*	Cavendish/L	Pingtung, Taiwan	-	+	-	-
ATCC76616	Fusarium wilt of lettuce/*F*. *oxysporum* f. sp. *lactucae*	Lettuce (*Lactuca sativa* L.)	California, USA	-	+	-	-
ATCC42006	Fusarium wilt of watermelon (FWW)/*F*. *oxysporum* f. sp. *niveum* (Fon)	Watermelon (*Citrullus lanatus* (Thunb.) Matsum & Nakai)/Vine (V)	Taiwan	-	+	-	-
ATCC42007	FWW/Fon	Watermelon/V	Taiwan	-	+	-	-
ATCC62940	FWW/Fon	Watermelon/Seed	Texas, USA	-	+	-	-
P33139	Bacterial wilt of eggplant/*Ralstonia solanacearum* (Rs)	Eggplant (*Solanum melongena* L.)/Stem	Taiwan	-	-	-	-
YJL-B001	Bacterial wilt of cucumber/Rs	Cucumber (*Cucumis sativus* L.)/V	Pingtung, Taiwan	-	-	-	-

^a^ The insulated isothermal polymerase chain reaction (iiPCR) method used for *Fusarium oxysporum* f. sp. *cubense* (Foc) race 4 detection was carried out in the R-tube^™^ within the POCKITTM Nucleic Acid Analyzer designed by GeneReach Biotechnology Corporation (Taichung City, Taiwan).

^b^ The conserved primer set ITS1/ITS4 was used to amplify and sequence the ~500-bp rDNA region used for the identification of the internal transcribed spacers 1 (ITS1), 5.8S rDNA, and ITS2 of the fungal pathogens used in this study.

^c^ The Foc race 4-specific primer set FocSc-1/FocSc-2 designed by Lin *et al*. [10] was used to confirm the specificity of the Foc race 4 iiPCR assay.

^d^ The Foc tropical race 4-specific primer set FocTR4-F/FocTR4-R designed by Dita *et al*. [15] was used to confirm the specificity of the Foc race 4 iiPCR assay.

### Primer and TaqMan probe design

The TaqMan probes and iiFoc primer used for the Foc race 4 iiPCR assays were designed on the basis of the recommended principles for iiPCR [20]. The iiFoc primer set iiFoc-1/iiFoc-2 and TaqMan probe iipFoc-1 (5’-6-FAM-ACCACGCGGATGAGATT-MGB-NFQ-3’) were designed according to the sequence of the Foc race 4-specific marker Foc_242_. The Foc_242_ marker has been confirmed to have high specificity for Foc race 4 [7,10]. The conserved primer set ITS1/ITS4 was used to amplify and sequence the ~500-bp rDNA regions, including the internal transcribed spacers 1 (ITS1), 5.8S rDNA, and ITS2 [28], for the purpose of identifying the isolates tested in this study. The other PCR-based identification techniques utilizing the Foc TR4-specific primer set FocTR4-F/FocTR4-R (designed by Dita et al. [15]) and the Foc race 4-specific primer set FocSc-1/FocSc-2 (designed by Lin et al. [10]) were used to confirm the specificity of the Foc race 4 iiPCR assay. All the sequences of the primer sets used in this study are listed in Table 2. The PCR conditions and protocols used for primer sets ITS1/ITS4, FocTR4-F/FocTR4-R, and FocSc-1/FocSc-2 were those described previously by White et al. [28], Dita et al. [15], and Lin et al. [10], respectively.

**Table 2:** Molecular markers and the corresponding primers used in this study.

Associated pathogens	Name of markers	Amplification primers		
		Names	Sequences (5'-3')	References
*Fusarium oxysporum* f. sp. *cubense* race 4	iiFoc_104_	iiFoc-1/iiFoc-2	CAGGGGATGTATGAGGAGGCTA/CGGAAACAGACTCTTGCCATTC	This study
All fungal pathogens	ITS1-5.8S-ITS2	ITS1/ITS4	TCCGTAGGTGAACCTGCGG/TCCTCCGCTTATTGATATGC	White *et al*. [28]
*F*. *oxysporum* f. sp. *cubense* race 4	Foc_242_	FocSc-1/FocSc-2	CAGGGGATGTATGAGGAGGCTAGGCTA/GTGACAGCGTCGTCTAGTTCCTTGGAG	Lin *et al*. [10]
*F*. *oxysporum* f. sp. *cubense* tropical race 4	FocTR4_463_	FocTR4-F/FocTR4-R	CACGTTTAAGGTGCCATGAGAG/CGCACGCCAGGACTGCCTCGTGA	Dita *et al*. [15]

### Sample DNA preparation

Dried fungal mycelium (100 mg), overnight-grown bacterial culture (1.5 ml), and field-infected banana tissues (300 mg) were frozen in liquid nitrogen and finely ground using a mortar and pestle. Genomic DNA (gDNA) was extracted according to Lin et al. [7], dissolved in a 0.1× TE buffer (1 mM Tris-HCl and 0.1 mM EDTA, pH 8.0), and stored at -20°C for further Foc race 4 iiPCR and PCR assays.

### Standard DNA preparation

The 404 bp RAPD marker (accession number EU379562) specific to Foc race 4 was published in an earlier study [7]. The specific primer sets FocSc-1/FocSc-2 (nt79-105/nt294-320) had been designed from the RAPD marker for rapid and specific detection of Foc race 4 by using PCR [7] and real-time PCR [10], respectively. To generate the standard template (named as pFoc_242_) used for iiPCR, the 242-bp DNA sequence amplified by the Foc race 4-specific primer set FocSc-1/FocSc-2 was gel-purified, cloned into pGEM^®^-T Easy vector (Promega Co, Madison, WI, USA), and sequenced. The copy number calculation of the standard template pFoc_242_ was based on the concentrations determined by a NanoDrop Lite spectrophotometer (Thermo Fisher Scientific Inc., Cleveland, OH, USA). The standard template pFoc_242_ was dissolved in a 0.1× TE buffer and stored at -20°C for further Foc race 4 iiPCR assay.

### Foc race 4 iiPCR assay

The Foc race 4 iiPCR was designed on the basis of the hydrolysis TaqMan probe-based POCKIT^™^ method described previously by Tsai et al. [22]. For this Foc race 4 iiPCR assay, each 50 μl iiPCR mixture contained tested gDNA, 1X Uni-ii HS Buffer (GeneReach Biotechnology Corp., Taichung, Taiwan), 0.5 mM of forward primer (iiFoc-1), 0.5 mM reverse primer (iiFoc-2), 200 nM of TaqMan probe (iipFoc-1) (Applied BioSystem, Life Technologies, Carlsbad, CA, USA), and 1 U of KAPA Taq DNA Polymerase (Kapa Biosystems, Boston, MA, USA). The iiPCR was carried out in the R-tube^™^ within the POCKIT^™^ Nucleic Acid Analyzer (GeneReach Biotechnology Corp., Taichung, Taiwan) following the instruction manuals. The fluorescent signal-to-noise ratios (signal_after_/signal_before_) collected by the POCKIT^™^ analyzer were converted automatically to positive (+ symbol) or negative (- symbol) according to the default S/N thresholds (S/N ratio ≥ 1.3) and then shown on the display screen of the POCKIT^™^ analyzer.

### Sensitivity and specificity determination

The specificity of the Foc race 4 iiPCR assay was tested by using the genomic DNA of plant pathogens isolated from banana plants, including Foc TR4, Foc ST4, Foc race 2, Foc race 1, and non-Foc, as well as those of other pathogens from non-banana plants, including *F*. *oxysporum* f. sp. *niveum* (Fusarium of watermelon), *F*. *oxysporum* f. sp. *lactucae* (Fusarium wilt of lettuce), and *Ralstonia solanacearum* (bacterial wilt). Serial dilutions of genomic DNA (ranging from 10^5^ to 1 fg) of Foc race 4 and standard template pFoc_242_ (ranging from 10^6^ to 1 copies) per reaction were subjected to sensitivity evaluation of the Foc race 4 iiPCR assay.

### Detection of field-banana samples

The Foc race 4 iiPCR assay was used to detect Foc in field-infected banana samples. Sampling criteria of infected banana pseudostems were adhered to according Lin et al. [10]. Specifically, necrosis covering less than 1/3 of the total area of a pseudostem, less than 2/3 but equal to or more than 1/3 of the total area, and equal to or more than 2/3 of the total area were recorded as mild, moderate, and severe symptoms, respectively. The field-infected banana pseudostems showing varying symptoms were individually sampled, surface-sterilized, and air dried. The surface-sterilized dried banana pseudostems were cut into 1 cm^2^ sections and put onto a Nash-PCNB agar medium for a plate-out assay. Simultaneously, a piece of the banana pseudostems (0.3 g) surrounding each section was used for DNA extraction according to Lin et al. [7]. The DNA samples (50 ng) of symptomatic and symptomless pseudostems were used for further Foc race 4 iiPCR.

## Results

### Optimization of Foc race 4 iiPCR assay

**Fig 2:**
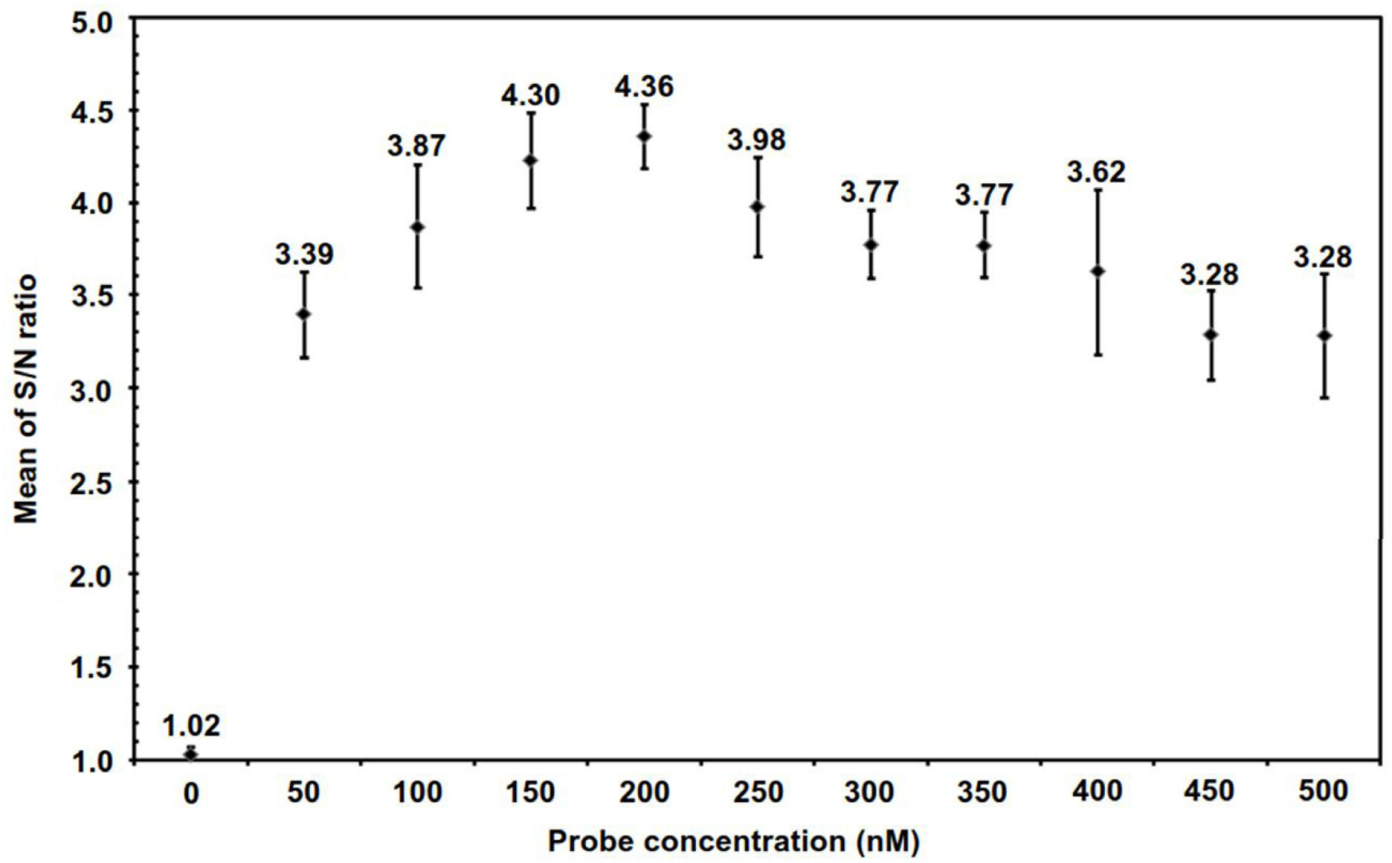
Optimization of TaqMan probe concentration for insulated isothermal PCR (iiPCR) assay of *Fusarium oxysporum* f. sp. *cubense* (Foc) race 4. Different concentrations (0, 50, 100, 150, 200, 250, 300, 350, 400, 450, and 500 nM) of probe were tested in the Foc iiPCR to evaluate the effects of TaqMan probe concentration on fluorescent signal production. The mean S/N ratio (fluorescent intensity_after_/fluorescent intensity_before_) of each reaction was plotted against the TaqMan probe concentration. Error bars represent the standard deviations from seven replicate reactions.

In order to develop a realizable iiPCR detection system for Foc race 4, the novel iiPCR primer set iiFoc-1/iiFoc-2 and TaqMan probe iipFoc-1 were developed according to the sequence of the Foc race 4-specific marker Foc_242_. Expected DNA bands were detected by the positive control ITS1/ITS4 in all isolates tested. The results of the Foc race 4 iiPCR detection were in agreement with the results produced using the other two PCR-based detection methods utilizing the Foc TR4-specific primer set FocTR4-F/FocTR4-R and the Foc race 4-specific primer set FocSc-1/FocSc-2 (Table 1). The concentration of TaqMan probe is known to influence the amplification and optical signal detection of iiPCR. Different concentrations (ranging from 0 to 500 nM) of the probe iipFoc-1 were tested to optimize the iiPCR for Foc amplification and signal detection with the POCKIT^™^ analyzer. The optimal iipFoc-1 probe concentration for the Foc race 4 iiPCR assay was 200 nM with a S/N ratio of 4.36 (standard deviation = 0.17) (Fig 2). Therefore, 200 nM of iipFoc-1 probe was the first priority choice for further Foc iiPCR purposes.

### Sensitivity and specificity evaluation of Foc race 4 iiPCR assay

A detection evaluation was performed to determine whether the Foc race 4 iiPCR assay was suitable for the detection of Foc race 4. Plant pathogenic isolates from banana plants, including Foc TR4, Foc ST4, Foc race 2, Foc race 1, *Alternaria alternata* (Alternaria speckle), *Botryosphaeria dothidea* (crown rot), *Cladosporium musae* (Cladosporium speckle), *Colletotrichum gloeosporioides* (anthracnose), *Cordana musae* (Cordana leaf spot), *F*. *sonani* (fruit rot), *F*. *subglutinans* (fruit rot), and as well as other pathogens from non-banana plants, including *F*. *oxysporum* f. sp. *lactucae* (Fusarium wilt of lettuce), *F*. *oxysporum* f. sp. *niveum* (Fusarium of watermelon), and *R*. *solanacearum* (bacterial wilt), were subjected to testing in order to assess the specificity of the Foc race 4 iiPCR assay using the POCKIT^™^ analyzer. The Foc race 4 iiPCR assay was positive for all of the isolates of Foc race 4 tested and negative for all the other pathogens of non-Foc race 4 (Table 1).

In addition, the results of the Foc race 4 iiPCR assay were consistent with those of the rDNA identification method using the universal primer set ITS1/ITS4 and with those of the PCR-based identification techniques using the Foc TR4-specific primer set (FocTR4-F/FocTR4-R) and Foc race 4-specific primer set (FocSc-1/FocSc-2) (Table 1). These data indicated that the Foc race 4 iiPCR assay offers a novel method for detecting Foc race 4 with high specificity.

**Fig 3:**
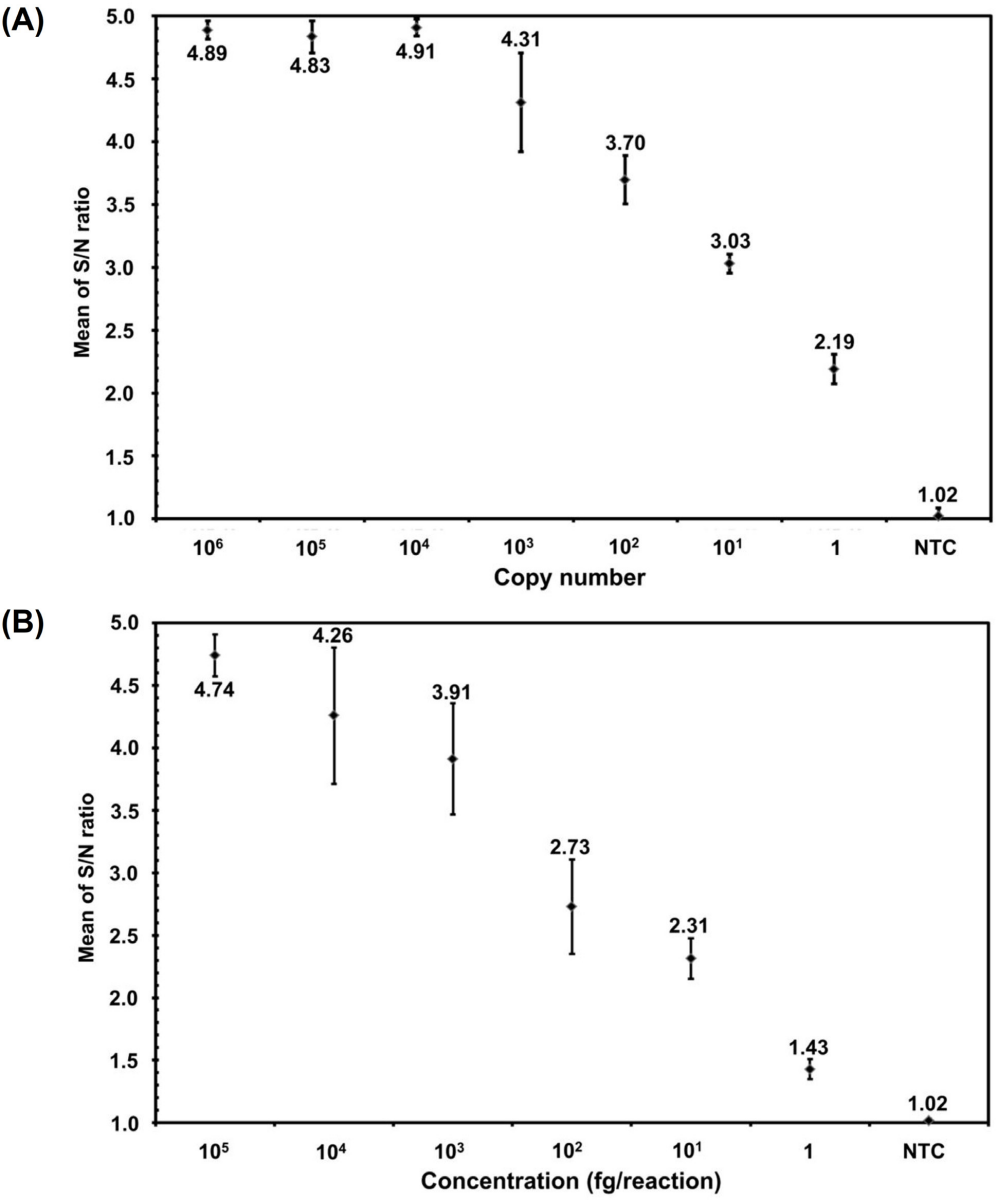
Sensitivity evaluation of Foc race 4 TaqMan probe-based insulated isothermal PCR (iiPCR) assay. Serial dilutions of (A) standard template pFoc_242_ (ranging from 10^6^ to 1 copies) and (B) *Fusarium oxysporum* f. sp. *cubense* (Foc) race 4 genomic DNA (ranging from 10^5^ to 1 fg) were subjected iiPCR assay. The S/N ratios (fluorescent intensity_after_/fluorescent intensity_before_) of Foc race 4 iiPCR assay were calculated. Mean S/N ratio of each reaction was plotted against standard template or Foc race 4 genomic DNA. Error bars represent the standard deviations from seven replicate reactions.

Serial dilutions of standard template pFoc_242_ (ranging from 10^6^ to 1 copies) and Foc gDNA (ranging from 10^5^ to 1 fg) were prepared to evaluate the sensitivity of the optimized Foc race 4 iiPCR assay. The Foc iiPCR data demonstrated that a standard template copy ranging from 10^6^ to 1 copies and a non-template-control (NTC) corresponded to average S/N ratios of 4.89±0.07 to 2.19±0.12 and 1.02±0.06, respectively (Fig 3A); and a Foc gDNA ranging from 10^5^ to 1 fg and an NTC corresponded to average S/N ratios of 4.74±0.17 to 1.43±0.08 and 1.02±0.02, respectively (Fig 3B). In addition, the sensitivity of the Foc race 4 iiPCR assay was not affected with 50 ng banana gDNA added to the reaction mixture containing standard template pFoc_242_ or the Foc gDNA (S1 Fig). The data indicated that we were able to obtain positive S/N results with high reproducibility even when the template was as low as 1 copy of standard template pFoc_242_ and 1 fg of Foc gDNA.

### Detection of field-infected banana samples by Foc race 4 iiPCR assay

A field detection evaluation was further performed to determine whether the Foc iiPCR was suitable for the detection of Foc race 4 in field-infected banana samples. For this purpose, varying symptomatic banana pseudostems were tested by the Foc iiPCR. The plate-out assay was used to confirm that the symptomatic bananas that were tested were Foc-infected while also indicating that the symptomless bananas that were tested were Foc-infected or Foc-free samples. Foc race 4 iiPCR assays showed average S/N ratios of 4.51±0.13, 4.56±0.05, 2.26±0.73, and 1.10±0.02 in severely symptomatic, moderately symptomatic, mildly symptomatic, and Foc-free pseudostems, respectively (Table 3). All the symptomatic banana pseudostem samples yielded positive Foc race 4 iiPCR results (Table 3), and the Foc race 4 iiPCR results agreed with the symptomatic detection and plate-out assay results, indicating that the Foc iiPCR is suitable for the detection of Foc race 4 in field-infected banana samples even if the infected bananas only exhibit mild symptoms.

**Table 3:** In planta detection of *Fusarium oxypsorum* f. sp. *cubense* race 4 in field-infected banana by insulated isothermal polymerase chain reaction (iiPCR) method.

Samples	Symptoms^a^	Plate-out assay^b^	Foc race 4 iiPCR results	
		No. positive/total results	No. positive/total results	Mean^c^ of S/N ratio^d^
Symptomatic pseudostems	Severe	6/6	6/6	4.51±0.13
Symptomatic pseudostems	Moderate	6/6	6/6	4.56±0.05
Symptomatic pseudostems	Mild	6/6	6/6	2.62±0.76
Foc-free pseudostems	No symptom	0/6	0/6	1.10±0.02
Amplification positive control (10^3^ copies of clone DNA)			6/6	4.65±0.18
Amplification no template control			0/6	1.07±0.03

^a^ Mild symptoms = less than 1/3 area of pseudostem necrosis; moderate symptoms = less than 2/3 but equal to or more than 1/3 area of pseudostem necrosis; severe symptoms = equal to or more than 2/3 area of pseudostem necrosis

^b^ A total of 24 varying symptomatic banana pseudostems were used for plate-out assay.

^c^ Mean ± standard deviation is presented from six replicate samples collected from six separate pseudostems.

^d^ S/N ratios were calculated from the values of the fluorescent intensityafter/fluorescent intensitybefore of the Foc race 4 iiPCR assay.

We collected a total of 63 banana pseudostem samples (47 Foc-infected pseudostems with mild symptoms and 16 symptomless but Foc-infested pseudostems) from 8 different fields in the Pingtung County for Foc race 4 iiPCR detection. These Foc race 4 iiPCR results for detecting the 47 pseudostems with mild symptoms were in agreement with those produced using the plate-out assay (Table 4) and the PCR-based identification method with the Foc race 4-specific primer set (Table 4). The detection rates of the Foc race 4 iiPCR and the PCR-based identification method were 16/16 (100%) and 9/16 (56.25%), respectively, when the 16 symptomless pseudostems were used as test samples. These data indicated that the Foc race 4 iiPCR assay with the primer set iiFoc-1/iiFoc-2 and TaqMan probe iipFoc-1 has high sensitivity for the in planta detection of Foc race 4; and the field-diagnostic iiPCR results seem to be supported by the symptomatic characteristics and plate-out assay results, as well as by those of the other PCR-based identification method.

**Table 4:** Comparison of Foc race 4 iiPCR and PCR-based identification for field detection.

Foc-infected pseudostems	Foc race 4 iiPCR			PCR-based identification		
	Positive	Negative	Total	Positive	Negative	Total
Mild symptom samples^a^	47	0	47	47	0	47
Symptomless samples^a^	16	0	16	9	7	16
Total	63	0	63	56	7	63

^a^ A total of 63 *Fusarium oxysporum* f. sp. *cubense* (Foc)-infected samples (including 47 pseudostems with mild symptoms and 16 symptomless but pathogen-infested pseudostems) collected from 8 different fields in Pingtung County were used to test the Foc race 4 iiPCR assay and the PCR-based identification using the Foc race 4 identification primer set FocSc-1/FocSc-2.

## Discussion

Foc race 4, which is the most virulent strain among all the races of Foc [5], is economically important as a destructive pathogen which causes Fusarium wilt on banana plants [10,12,15]. Thus far, Foc cannot be controlled with fungicides [15] because it can produce thick-walled chlamydospores which are highly resistant to chemical fumigation [2] and can survive in the infected soil as resting spores for numerous years [1,3–5]. Susceptible varieties cannot be successfully replanted in Foc-infected soil, while commercial banana cultivars resistant to Foc race 4 and TR4 have also not been extensively planted [10]. Therefore, refraining from planting any susceptible banana cultivars in Foc-infected fields, as well as preventing the introduction and dissemination of Foc from diseased to healthy banana plants as much as possible, are the top priorities in disease management strategies of FW.

Thus far, a variety of PCR-based methods which can be used to detect Foc race 4 have been made available [7]. However, gel electrophoresis, which is the standard procedure for analyzing PCR products, is relatively time-consuming. In this study, a TaqMan probe-based race 4 iiPCR assay for rapid in planta detection of Foc race 4 was developed. TaqMan probes derive fluorescence signals from the hydrolysis of a probe by 5’ to 3’ exonuclease activity of Taq polymerase, resulting in a significant increase in S/N ratios (signal intensity_after_/signal intensity_before_). The iiPCR fluorescent signals are detectable through use of the relatively simple iiPCR device, the POCKIT^™^ Nucleic Acid Analyzer. The major advantage of this assay is that, compared with conventional PCR, the Foc race 4 iiPCR assay does not require separate equipment to perform PCR and signal detection.

In this study, a Foc race 4 iiPCR assay was developed using the primer set iiFoc-1/iiFoc-2 and TaqMan probe iipFoc-1 according to the Foc race 4-specific marker OPA02_404_ (referred to as accession number EU379562) published by Lin et al. [7]. The sequence EU379562 is identical to the other sequence of a *F*. *oxysporum* f. sp. *cubense* isolate FOC-FT marker (referred to as accession number EF155535). The sequence EF155535 had been used for developing a Foc race 4 LAMP assay by Li et al. in 2013 [16]. The DNA marker should be specific to Foc race 4 and previously was tested with over a hundred isolates of *F*. *oxysporum* [7,10,16]. Hence, we used the Foc race 4-specific marker for developing the Foc race iiPCR assay. The optimal iipFoc-1 probe concentration for the Foc race 4 iiPCR assay was determined (Fig 2). A variety of diseases affecting banana plants were used for detection evaluation to determine whether the Foc iiPCR assay was suitable for the clinical detection of Foc race 4. The results of the clinical Foc iiPCR detection were supported by those of two reference PCR-based detection methods (Table 1).

The Foc iiPCR is a simple and accurate method for detecting Foc race 4 in a variety of symptomatic pseudostem samples (Table 3). High field-detection reproducibility in Foc race 4 detection by the Foc race 4 iiPCR assay was observed among the results generated from the random 63 field-collected pseudostems with mild symptoms or symptomless. These field-detection reproducibility results were supported by those of traditional plate-out assays and molecular detection assays with the reference primer sets (Table 4).

Several studies have reported the use of the real-time PCR [10], LAMP [16], and RealAmp [18] methods to sensitively detect Foc race 4. The results of the sensitivity evaluation showed that the detection limit of the Foc race 4 iiPCR assay was comparable to that of the published detection methods of Foc race 4. Specifically, the minimum detection concentrations required for our iiPCR, the real-time PCR [10], and LAMP [16] were 1 fg, 1 fg, and 10 fg of Foc race 4 genomic DNA, respectively. The detection sensitivity of the RealAmp was 3.82 × 10^3^ copies of standard DNA in artificially infested soil [18], a sensitivity value that is difficult to compare with that of the Foc iiPCR assay.

In conclusion, to our knowledge, this is the first study to report the detection of phytopathogenic fungi using a TaqMan probe-based iiPCR assay. We have developed an efficient and sensitive Foc race 4 iiPCR assay for the detection of Foc race 4. The Foc race 4 iiPCR developed in this study provides an alternative to conventional PCR, real-time PCR, LAMP, and RealAmp assays in the detection of Foc race 4. This Foc race 4 iiPCR assay has the potential to serve as a rapid, specific, and sensitive tool for the routine in planta detection of Foc race 4 for the banana.

## Supporting Information

S1 Fig Effect of banana genomic DNA (gDNA) on the detection sensitivity of Foc race 4 TaqMan probe-based insulated isothermal PCR (iiPCR) assay. Fifty ng banana gDNA was added to Foc race 4 iiPCR reaction mixture containing (A) pFoc_242_ standard template (ranging from 10^6^ to 1 copies) or *Fusarium oxysporum* f. sp. *cubense* (Foc) race 4 gDNA (ranging from 10^5^ to 1 fg) for sensitivity evaluation. The S/N ratios (fluorescent intensity_after_/fluorescent intensity_before_) of Foc race 4 iiPCR assay were calculated. Mean S/N ratio of each reaction was plotted against standard template or Foc race 4 gDNA. Error bars represent the standard deviations from seven replicate reactions.
(PDF)
