# Supplementary material for: Development of a TaqMan Probe-Based Insulated Isothermal Polymerase Chain Reaction (iiPCR) Assay for Detection of Fusarium oxysporum f. sp. cubense Race 4
Source: PLoS One. 2016 Jul 22;11(7):e0159681. doi: 10.1371/journal.pone.0159681 (PMC4957775; doi:10.1371/journal.pone.0159681)
Supplement: S1 Fig — Fifty ng banana gDNA was added to Foc race 4 iiPCR reaction mixture containing (A) pFoc242 standard template (ranging from 106 to 1 copies) or Fusarium oxysporum f. sp. cubense (Foc) race 4 gDNA (ranging from 105 to 1 fg) for sensitivity evaluation. The S/N ratios (fluorescent intensityafter/fluorescent intensitybefore) of Foc race 4 iiPCR assay were calculated. Mean S/N ratio of each reaction was plotted against standard template or Foc race 4 gDNA. Error bars represent the standard deviations from seven replicate reactions. (PDF) [file pone.0159681.s001.pdf]

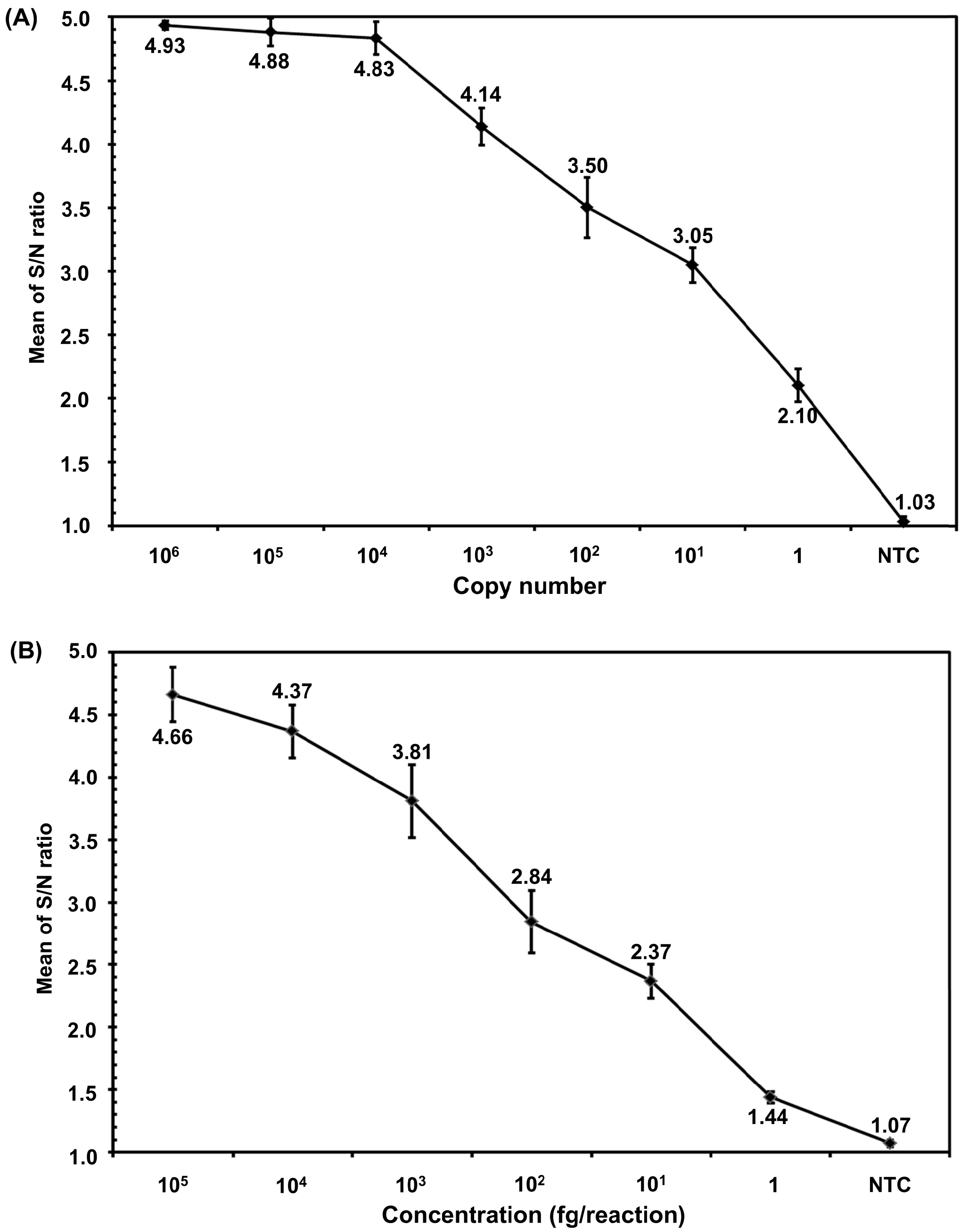

S1 Fig. Effect of banana genomic DNA (gDNA) on the detection sensitivity of Foc race 4 TaqMan probe-based insulated isothermal PCR (iiPCR) assay.

Fifty ng banana gDNA was added to Foc race 4 iiPCR reaction mixture containing (A) $pFoc_{242}$ standard template (ranging from 10^6 to 1 copies) or *Fusarium oxysporum* f. sp. *cubense* (Foc) race 4 gDNA (ranging from 10^5 to 1 fg) for sensitivity evaluation. The S/N ratios (fluorescent $intensity_{after}$/fluorescent $intensity_{before}$) of Foc race 4 iiPCR assay were calculated. Mean S/N ratio of each reaction was plotted against standard template or Foc race 4 gDNA. Error bars represent the standard deviations from seven replicate reactions.
